# Deterministic Mechanical Model of T-Killer Cell Polarization Reproduces the Wandering of Aim between Simultaneously Engaged Targets

**DOI:** 10.1371/journal.pcbi.1000260

**Published:** 2009-01-09

**Authors:** Mun Ju Kim, Ivan V. Maly

**Affiliations:** Department of Computational Biology, University of Pittsburgh School of Medicine, Pittsburgh, Pennsylvania, United States of America; University of Virginia, United States of America

## Abstract

T-killer cells of the immune system eliminate virus-infected and tumorous cells through direct cell–cell interactions. Reorientation of the killing apparatus inside the T cell to the T-cell interface with the target cell ensures specificity of the immune response. The killing apparatus can also oscillate next to the cell–cell interface. When two target cells are engaged by the T cell simultaneously, the killing apparatus can oscillate between the two interface areas. This oscillation is one of the most striking examples of cell movements that give the microscopist an unmechanistic impression of the cell's fidgety indecision. We have constructed a three-dimensional, numerical biomechanical model of the molecular-motor-driven microtubule cytoskeleton that positions the killing apparatus. The model demonstrates that the cortical pulling mechanism is indeed capable of orienting the killing apparatus into the functional position under a range of conditions. The model also predicts experimentally testable limitations of this commonly hypothesized mechanism of T-cell polarization. After the reorientation, the numerical solution exhibits complex, multidirectional, multiperiodic, and sustained oscillations in the absence of any external guidance or stochasticity. These computational results demonstrate that the strikingly animate wandering of aim in T-killer cells has a purely mechanical and deterministic explanation.

## Author Summary

Beyond the more widely known molecular recognition of antigen, specificity of the cellular immune response relies on the precise orientation of immune cells toward infected and tumorous cells. We studied the mechanics of the structural orientation of T-killer cells (a type of immune cells) to their immunological targets. One of the most remarkable features of this process as seen under the microscope is the apparent “wandering of aim”: instead of pointing steadily at the intended target, the killing apparatus inside the T-killer cell can wave around. When two targets are engaged simultaneously, the killing apparatus in the T cell can repeatedly oscillate between the two. It might appear that the origin of this strikingly animate behavior should lie in stochasticity of the underlying mechanism. Our numerical model, however, was able to reproduce the complex, continuing motion in spite of the fact that the model was purely deterministic. This result suggests that deterministic quantitative explanations and supporting experimental evidence can be sought in the other cases of extremely complex cell motility that give the microscopist an acute sense that the object is alive.

## Introduction

The high specificity of the immune response depends in large measure on direct cell-cell interactions. An example is the interaction of a T-killer lymphocyte with a tumor cell, or with a cell that has been infected and is producing new viral particles. It is generally accepted (e.g., ref. [1]) that the T-killer cell patrols the tissue, comes in contact with the abnormal cell, recognizes the specific antigen on its surface, develops firm contact with the target cell, and releases toxic compounds in its direction. The directionality of the release, which makes the killing efficient and spares the bystander cells, is arguably as important as the precise molecular recognition of the antigen for the specificity of the immune response [2],[3].

The killing apparatus in T cells is structurally assembled around the centrosome, the organelle in which the microtubule fibers of the cytoskeleton are anchored. Experiments suggest that the killing apparatus may be positioned next to the target cell by molecular motors. According to this hypothesis, dynein motors anchored at the T cell interface with the target “reel in” the centrosome by pulling on microtubules that pass over the interface [4],[5]. Surprisingly, large fluctuations of the centrosome next to the cell-cell interface have been observed, as well as fluctuations between interfaces with simultaneously engaged targets [4].

Is the pulling mechanism biophysically plausible? And what is the nature of the apparent wandering of aim in T-killer cells? Here we show by means of biomechanical modeling that the pulling mechanism is indeed capable of bringing about the functional orientation of the centrosome in a range of conditions. Our analysis also predicts substantial and verifiable limitations of this mechanism. Our calculations show that the complex fluctuations are an intrinsic property of this mechanism and of the T-cell structure, in the absence of any stochasticity or external guidance, suggesting a deterministic mechanical explanation for one of the most “animate” cell behaviors.

## Results/Discussion

### Critical Assumptions

**Figure 1:**
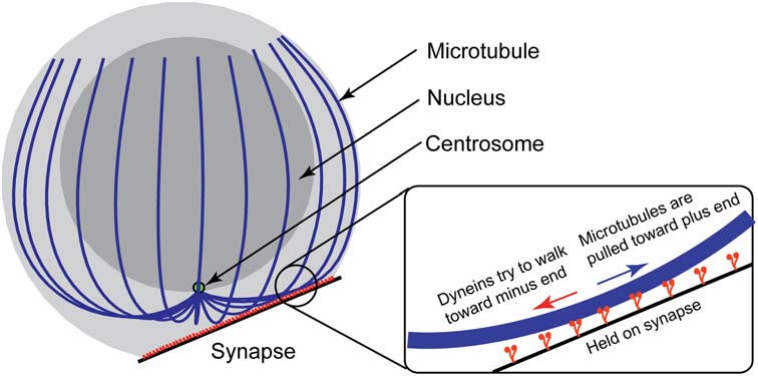
Schematic of the model. The cartoon depiction of the dynein motor molecules (red) is for visualization purposes only. Individual dynein molecules are not modeled computationally, only the pulling force they produce. Microtubule thickness is greatly exaggerated in the diagram. The centrosome (green) is merely a marker in the diagram; the centrosome in the model is identified with the common anchoring point of the microtubules.

From the experimental videos [4] we obtain the following idealizations to set up our numerical model (refer to the diagram in Figure 1). The cell outline consists of an unattached round part and of a flat part which is attached to the target cell (called synapse, or synaptic plane). The large nucleus is coupled to the aster of microtubules converging near its surface, and the mobility of both is constrained by the cell outline. Microtubules slide along the cell outline in the areas of contact with the targets. This active sliding–specified in more detail below–drives all movements that are observed. The movements are opposed by microtubule bending elasticity and by viscous drag in the cytoplasm. This condition completes the physical specification of our model; for its exact numerical implementation, refer to the Methods section.

The active microtubule sliding in the model is meant to represent the action of cortically anchored molecular motors. Idealizing what should happen when microtubules come in contact with the cell cortex on which motor molecules are anchored [4],[5], we assume that the unit length of the contacting part of the microtubule will experience a constant tangential force. The mechanical property of the synapse with respect to a microtubule is therefore characterized in our model by a one-dimensional force density (units of force per length). It is additionally assumed that the force exerted on the microtubule is directed, along the local tangent to the microtubule, to the end of the microtubule that is free (not attached to the centrosome). This end is commonly referred to as the plus end of the microtubule. The direction of the force so exerted on the microtubule is the intrinsic property of the dynein-type molecular motors that have been implicated in T cell polarization [5]. In the more commonly considered situation of vesicular transport, dynein motors ferry intracellular cargo to the so-called minus end of the microtubule (the end that is anchored at the centrosome). Considering the action and reaction forces, when the intracellular vesicle is moved along the microtubule to the minus end, the force exerted by dynein on the microtubule is directed to the plus end. We assume that the force direction is the same also in the case where the dynein-type motor is anchored at the inner surface of the cell outline in the synapse area. The arrangement of motors on this surface can be envisioned as entirely random (uniform and isotropic). This is the implication behind our cell-level model assumption that the direction of the force acting on a microtubule depends only on the direction in which the microtubule passes over the inner surface of the synapse. Indeed, one can envision motor molecules that can pivot on their cortical attachments and will therefore be aligned by their very interaction with a microtubule. Alternatively, motors may be randomly and stably oriented, and only the ones with a matching orientation will engage with the microtubule passing over the synapse in a certain direction. In both cases the pulling force density experienced by the microtubule will be a constant, and the resulting force will be tangential to the microtubule. The effectively isotropic arrangement of motors is considered here merely as the simplest possibility in the absence of empirical data on what an anisotropic arrangement could be like. The model assumption of the constant pulling force density also stipulates that, in molecular terms, there should always be a sufficient number of individual motor molecules in contact with the microtubule. Then the pulling on that microtubule can be processive (continuous), whether the individual motors are processive or not: When some motor molecules disengage, others engage, and the average pulling force is continuously exerted. We would like to emphasize that all considerations regarding the motors are not part of our quantitative model per se but are plausible molecular interpretations of the actual model assumption of the constant density and tangentiality of the pulling force.

### Reorientation

**Figure 2:**
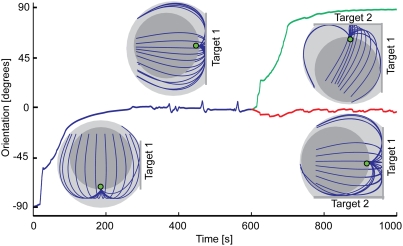
Centrosome reorientation in the model. Dynamics of the centrosome orientation in a T cell developing sequentially two synapses is shown. The insets are computer-generated snapshots of the actual numerical model cell. The graphic conventions are the same as in Figure 1. Flattened interfaces with target cells are also depicted. The centrosome is initially pointing down (orientation −90°), and the first synapse develops on the cell equator (orientation 0°). The evolution of the centrosome orientation with time is shown by the blue plot. Note the oscillations following the stabilization of the equatorial position of the centrosome. After this (at *t* = 10 min) the model cell is set to develop the second synapse. In one version of the simulation (green plot), the second synapse develops on the top of the cell, and the centrosome rapidly migrates to it. In the alternative branch of the simulation (red plot), the second synapse develops on the bottom of the cell. In this case, the centrosome does not leave its position near the middle of the first synapse (red line). Both of the alternative centrosome positions seen at the end of this graph persist for much longer than plotted. Pulling force density, 40 pN/µm; microtubule length, 16 µm; effective cytoplasm viscosity, 2 pN s/µm^2^.

Figure 2 and Video S1 show a simulation where the centrosome is initially oriented at 90° to the developing cell-cell interface. This orientation is the likeliest if the spherical T cell comes in contact with the target surface entirely at random. This is so for the following reasons. Centrosomes facing any point around a circumference on the T cell surface, which circumference is parallel to the forming synapse, will all have an identical angular separation from the synapse. Indeed, which way the centrosome is facing around the axis perpendicular to the synapse, is of no consequence for the magnitude of the reorientation that is required to bring the centrosome into the functional orientation toward the synapse. Such a circumference corresponding to the identical orientation with respect to the synapse will be the longest, when the angular separation of the centrosome from the synapse is 90°. Random orientation of the T cell cytoskeleton in three dimensions would mean that the centrosome is equally likely to point towards any small area on the spherical outline of the T cell body. The longest circumference then corresponds to the likeliest orientation with respect to the synapse, which is therefore 90°. Our model reproduces the observation that the centrosome becomes reoriented to the interface. Interestingly, stabilization of the centrosome orientation in the model is soon followed by development of pulse-like oscillations of the centrosome position (Figure 2). The oscillations are in agreement with the experimental observations [4], and are analyzed in more detail below. An interesting prediction of the model is that the long-range reorientation also results in an arrangement of microtubules that is very asymmetrical. On the side of the microtubule aster that was leading during the reorientation movement (i.e., on the side next to which the synapse initially developed), a relatively tight “bundle” of microtubules is formed. The bundle is separated by a distinctive gap from the microtubules that were trailing. There exists a published three-dimensional experimental image of an *early* T cell-target cell conjugate (Figure 6a in ref. [4]) that may arguably show a similar gap. However, the gap formation has not been specifically investigated experimentally, and therefore remains a prediction to be verified. For the verification it will be important that in the model the gap is a transient feature seen after reorientation, not a static-equilibrium configuration. (Available images of fully established T cell-target cell conjugates, e.g., in ref. [4], show only a comparatively symmetric structure.) The induced asymmetry in the model aster should be responsible for the ratchet-like behavior of the microtubule cytoskeleton, which is predicted by our model when the T cell develops a second synapse. The centrosome readily reorients by another 90° in the same direction as it did the first time, but does not reorient in the opposite direction (Figure 2). In view of this, another testable prediction can be made regarding the experimentally observed oscillations of the centrosome between two synapses: The cortical-pulling mechanism does not permit reversible intersynaptic oscillations in cases where the centrosome undergoes a large reorientation to the synapse that is the first to develop. Before embarking on the analysis of the mechanical conditions that do permit the intersynaptic oscillations, the capacity of the pulling mechanism for achieving the functional polarity of the T-cell cytoskeleton needs to be outlined more systematically.

The orientation of the centrosome is described here using an angular measure. The rounded outline of the T cell makes the angular measures and the terms “orientation” and “reorientation” convenient. It also makes the centrosome trajectory during the long-range reorientation look at least partly like an arc. To show as much of this movement as two-dimensional representation can convey, we chose throughout our paper to show reorientation in figures and videos from such an angle that the line of sight is directed along the axis of the arc. From any other angle, the same movement would appear only less arc-like, and more “vectorial”. In this sense, we feel that our model is compatible with the vectorial description of translocation in experiments [4].

The movements in our model are, strictly speaking, a superposition of the movements caused by pulling and of movements caused by the deformation of the cell outline in the beginning of each simulation. Simulations in which pulling force density was set to zero (Figure S1) show, however, that the “passive” component is small, usually not exceeding several degrees of centrosome rotation. Thus, in the framework of the present model, achieving any specific centrosome position, such as next to the synapse (or at the rear in a migrating T cell), requires the active pulling force.

**Figure 3:**
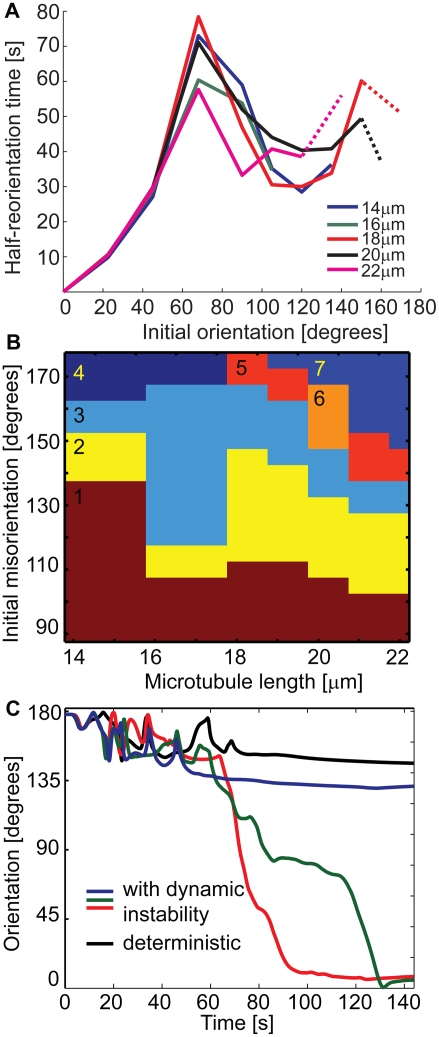
Quantitative analysis of centrosome reorientation. (A) The time it takes the model centrosome to reorient by one-half of the initial angular separation, as a function of this initial separation, plotted for the indicated values of the microtubule length. The segments of the broken lines connect the points corresponding to the actual simulation results; where the segments are dashed, it indicates that they connect two data points between which a data point is missing because the half-reorientation could not be achieved. Pulling force density, 40 pN/µm; effective cytoplasm viscosity, 2 pN s/µm^2^. (B) Qualitatively different predictions obtained with the different microtubule length and initial angular separation between the centrosome and the middle of the synapse. Regions in the two-dimensional parameter space are color-coded and numbered. In region 1, the complete reorientation is achieved. In region 2, the reorientation is “jammed” at around 30° of remaining angular separation. In region 3, the reorientation is “jammed” at the characteristic angular separation of 100°. In region 4, reorientation does not commence because the microtubules are too short to contact the synapse. In region 5, complete reorientation is achieved after a catastrophic stability loss of the “locked” configuration of antiparallel microtubules overlapping at the synapse. In region 6, the same happens but the final reorientation is as incomplete as in region 2. In region 7, the “locked” overlapping configuration is stable and no reorientation occurs. Pulling force density, 40 pN/µm; effective cytoplasm viscosity, 2 pN s/µm^2^. (C) Effect of microtubule dynamic instability on the stability of the “locked” configuration such as predicted in region 7 of (B). Angular position of the centrosome is plotted vs. time as predicted by the purely deterministic model analyzed throughout the paper (black curve) and with an additional assumption of stochastic microtubule dynamic instability (colored curves). The three stochastic simulations are independent (in the sense of pseudo-random number generation on a computer) repetitions of a simulation which was otherwise set up the same way as the deterministic one. The angle plotted is defined as the angle formed by the lines drawn from the nucleus center to the centrosome and to the middle of the synapse. The deterministic prediction is that the centrosome, having started facing the opposite side of the cell from the synapse, will not be able to reorient to the synapse. The stochastic predictions differ between runs: one is similar to the deterministic prediction, in the other two the centrosome was able to reorient. Pulling force density, 40 pN/µm; microtubule length (starting microtubule length in stochastic simulations), 21.5 µm; effective cytoplasm viscosity, 2 pN s/µm^2^.

Given the quasi-exponential kinetics of the reorientation to the target, i.e., one characterized by a rapid beginning followed by a slow stabilization at the final position (Figure 2), it is appropriate to measure the rapidity of the reorientation by the time it takes to reorient by one-half of the angle that separated the initial and the functional orientations of the centrosome. This is analogous, for example, to the widely used half-recovery time in photobleaching experiments. The half-reorientation time achieved by the dynein-pulling mechanism in our model is plotted in Figure 3A vs. the initial misorientation of the centrosome, i.e., vs. the angular separation of the initial centrosome orientation and the middle of the forming synapse. This plot is essentially the structural challenge – kinetic response curve for the T cell polarization driven by the cortical dynein. It shows that for the comparatively small required reorientations, up to about 70°, the rise of the response time is nonlinear: the movement induced is actually the slower the larger reorientation is needed. This can be attributed to the spatial separation of the microtubules diverging from the centrosome. As a result, a synapse of the given size that is formed farther away will be contacted by fewer microtubules, and the integral force exerted on the microtubule cytoskeleton by such a synapse will be smaller. Figure 3A further shows that this dependence breaks down for even larger “challenges”: Between about 70 and 110° of the initial separation of the synapse from the centrosome, the half-reorientation time actually goes down with the increasing reorientation range. This can be attributed to the advantages of the tighter contact of microtubules with the pulling surface. The microtubules can therefore experience a larger pulling force. This apparently becomes the overriding factor in this range of initial misorientations. (Notice in the initial, mechanically relaxed cell structure shown in the first inset in Figure 2 that the more distal parts of the microtubules are straighter and potentially better aligned with a synapse that can form next to them than the highly curved proximal parts can be.) The challenge-response plot in Figure 3A shows further that for initial misorientations that are larger still, the half-reorientation time displays a tendency to rise and fall once more, but the kinetics becomes much more dependent on the microtubule length, and the half-reorientation may not then be achieved at all.

The complexity of outcomes reveals the limitations imposed by the basic cell structure on the functional capacity of the pulling mechanism. The chart of the simulation outcomes (Figure 3B) shows that the functionally required reorientation up to about 100° can be completed by the cell with microtubules of any plausible length (Figure 3B, region 1). However, as the initial separation of the centrosome and the synapse increases, the microtubule cytoskeleton is predicted to become jammed at certain positions without reaching the fully functional orientation (regions 2 and 3). This is apparently due to limits to the movement of the microtubule aster in the space between the nucleus and the outline of the cell. For certain microtubule lengths and initial orientations (region 4), the microtubules are simply too short to contact the synapse and initiate any movement. Interestingly, examination of the boundary between regions 1 (“success”) and 2 (“jammed”) shows that making microtubules longer can actually create impediments to complete reorientation, when the movement could otherwise commence. The most interesting in this regard are the predictions for the largest initial misorientations of the centrosome with respect to the forming synapse, such as near 180°, which is commonly hypothesized to be the case in vivo (e.g., ref. [1]). If the microtubules are long enough to reach such a synapse, they will also likely to be long enough to overlap there in the anti-parallel fashion. The model shows that in this case, the pulling will lock the microtubule system in place (with microtubules wound tightly around the nucleus), rather than reorient it. This can happen even if the synapse is quite far from being symmetrically opposite the centrosome, provided only that the microtubules are long enough to overlap at the synapse (Figure 3B, region 7). However, for certain microtubule lengths and initial orientations (regions 5 and 6), the locking, although it may initially appear stable, is resolved through a catastrophic loss of stability, and reorientation can then commence. Interestingly, the comparatively violent loss of stability may make possible final reorientation that is complete, even though this region in the parameter space (region 5) is beyond the zone where functional orientation was already impossible in the absence of any locking (region 3). The predicted variability of the dynein-driven cytoskeleton polarization in T cells, depending on the exact initial orientation and individual cell structure, appears very life-like and demands experimental testing.

Additional simulations where dynamic instability [6],[7] was included show that the jamming may be overcome if the microtubule length is not constant but undergoes stochastic fluctuations. Our model predicts that due to the very stochastic nature of dynamic instability, the jamming may be overcome in some cells and not in others (Figure 3C). Statistically, therefore, dynamic instability of microtubules has the capacity to facilitate reorientation driven by pulling.

The mechanically dead-locked state with the non-functional orientation of the centrosome has not been experimentally documented. This suggests three possibilities: (1) the specific initial conditions that lead to it in the model (region 7 in Figure 3B) are not encountered in reality; (2) the pulling mechanism is not the correct mechanism, or should be translated substantially differently into quantitative model assumptions; (3) the pulling mechanism is complemented by other mechanisms in reality. The first possibility is likely because the locking is predicted only in a small fraction of the feasible parameter space (region 7 in Figure 3B). The second possibility is less likely, because the other predictions reproduce a number of striking experimental observations. The third possibility is highly likely; in particular, our simulations suggest that dynamic instability of microtubules is one such additional mechanism that has the capacity to resolve the locking. Disintegration of microtubules under load is another possibility in this regard that our present model does not consider. It is however made less likely by the fact that excessive bending is not seen in our simulations. The axial stress induced by the pulling force in our simulations is likely to be withstood. Measurements suggested that microtubules have mechanical properties resembling Plexiglas [8]. From this, M. W. Berns and colleagues [9] estimated that, although the yield strength of a microtubule is not known, it can be similar to that of polymethylmethacrylate, 40–70 MPa. Considering the cross-section of microtubules 25 nm in diameter, we conclude that microtubules should be able to bear the tensile loads encountered in our model (up to ∼100 pN) without structural disintegration.

### Intra-Synaptic Oscillations

**Figure 4:**
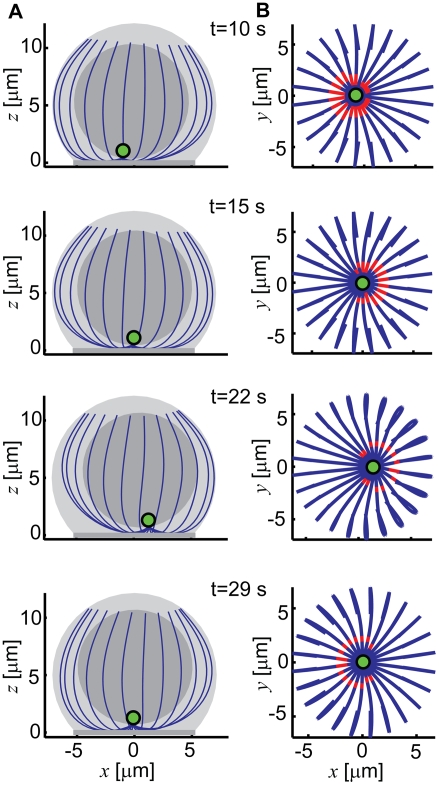
Oscillations of the centrosome within the synaptic area. (A) Graphs of the model cell structure at the indicated time points. (B) The oscillating microtubule system shown in projection onto the synaptic plane. The parts that are in contact with the synaptic surface and are experiencing the pulling are highlighted in red. Pulling force density, 20 pN/µm; microtubule length, 16 µm; effective cytoplasm viscosity, 2 pN s/µm^2^.

**Figure 5:**
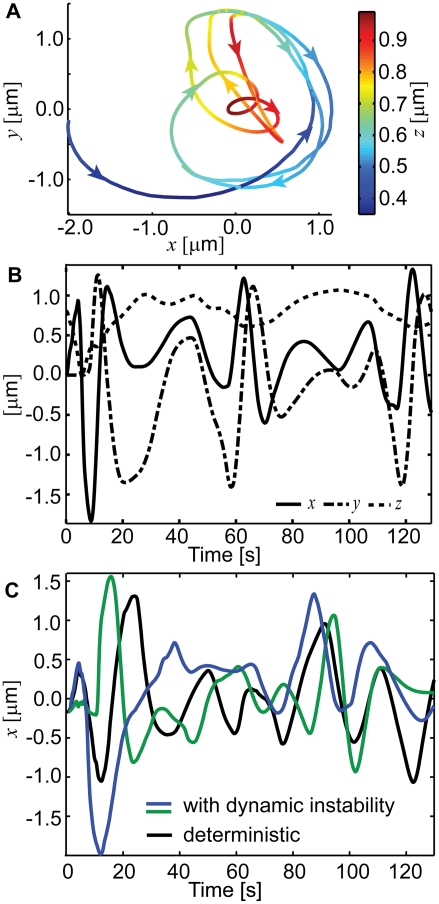
Typical trajectories of centrosomes oscillating within a synaptic area. (A) A centrosome trajectory in projection onto the synaptic area, with color denoting the height above it and arrows, the direction. The directions of axes are as indicated in Figure 4. Pulling force density, 40 pN/µm; microtubule length, 16 µm; effective cytoplasm viscosity, 2 pN s/µm^2^. (B) Positions of the centrosome along the two horizontal axes and its vertical position plotted vs. time. Note the phase shift between the oscillations along the *x* and *y* axes that leads to gyrations visible in (A), and apparent beats. Pulling force density, 40 pN/µm; microtubule length, 16 µm; effective cytoplasm viscosity, 2 pN s/µm^2^. (C) Effect of microtubule dynamic instability and of an annular shape of the pulling surface on the pattern of oscillations. Position of the centrosome is plotted vs. time as predicted by the purely deterministic model with the disk-shaped pulling surface, as analyzed throughout the paper (black curve), and with stochastic microtubule dynamic instability and annular pulling surface (colored curves). The two stochastic simulations are independent in the sense of pseudo-random number generation on a computer. The stochastic predictions differ between runs but preserve the characteristic features of the deterministic one. Pulling force density, 20 pN/µm in the deterministic simulation and 36 pN/µm in the stochastic simulations. Microtubule length (starting microtubule length in stochastic simulations) was 16 µm, effective cytoplasm viscosity, 2 pN s/µm^2^.

Returning to the analysis of the purely deterministic effects of pulling (without incorporating the dynamic instability of microtubule length in the model), we analyzed further the mechanism of the deterministic mechanical instability of the centrosome position that followed the long-range reorientation. Video S2 shows a generic case of oscillations developing after the functional position of the centrosome next to the synapse is reached. It was found that oscillations develop in the model even if the synapse is formed next to the initial location of the centrosome. An otherwise insignificant tilt of the synapse (such as 2°) will determine the initial phase of the oscillations in our deterministic model. Engagement of the microtubules with the pulling surface causes the model centrosome to greatly “overshoot” and to continue moving beyond the center point of the interface. It eventually stops and begins the reverse motion, again approaching the center point and again overshooting (Figure 4A). The oscillations may persist without noticeable systematic changes over at least 1 h of simulated physical time. Typically it appears that there are overlapping and interfering periodic motions (Figure 5B). Also, oscillatory movements that are mostly tangential to the model cell-cell interface occur simultaneously with oscillatory movements that are orthogonal to it (Figure 5B). Gyrations (looping motions parallel to the interface) can also be discerned in the complex trajectory of the model centrosome (Figure 5A).

To determine the impact dynamic instability of microtubules [6],[7] and ring-shaped distribution of pulling motors [5] might have on the deterministic oscillations, we performed additional simulations that incorporated these structural and kinetic details. The dynamic instability was modeled as in the simulations described above (Figure 3C). The ring-shaped distribution of dynein was modeled by assuming that only the annulus between 0.225*R* and 0.775*R*, where *R* is the synapse radius, could exert pulling force on the microtubules. (The annulus is shown in Video S3.) Results show that incorporation of these kinetic and structural details does not dramatically affect the oscillations predicted by the simple deterministic model (Figure 5C and Video S3). Overall our results suggest that although dynamic instability of microtubules and ring-shaped distribution of dynein may influence the exact trajectory of the centrosome in living cells, they need not be the root cause of the oscillations, nor do they necessarily have a large impact on the oscillation pattern. It is interesting that, as can be seen in Figure 4B and Video S4, when the pulling surface is assumed to be a disk, the area actually contacted by the microtubules is nonetheless a ring, due to how the microtubules bend against the synapse. This may explain the absence of a significant effect of the assumption of the shape of the pulling area (ring or disk) on the oscillations dynamics. Also, the movement of the centrosome to the edge of the synaptic area in the model is restricted by bending of microtubules against the sides of the cell, as discussed above. A similar effect restricting the centrosome movement is predicted to arise, in the case of the ring-shaped pulling area, from the reversion of polarity of microtubules contacting the pulling annulus as the centrosome crosses it. It is tempting to speculate that real T cells [4],[5] may arrange their cortical motors in the ring-shaped areas not to waste any in areas not contacted by microtubules. In the rest of our analysis we refer only to the case of purely deterministic and structurally simplified modeling that does not incorporate the dynamic instability or the ring-shaped distribution of dynein.

As regards the origin of the deterministic oscillations and of the repeated overshooting which are exhibited by the centrosome, it is important to point out that inertia plays no role in intracellular movements due to the prevailing near-zero Reynolds number conditions. In fact, like in models for comparable types of intracellular movements (e.g., refs. [10]–[12]), there is no mass in our mechanical model. Also, the model is strictly deterministic, and therefore the deflections from the middle position of the centrosome are not due to molecular stochasticity. Close inspection of the model reveals that when the centrosome passes the middle point during oscillations, the microtubule aster shows significant asymmetry. This asymmetry is reversed when the centrosome passes the middle point the next time (Figure 4B and Video S4). Moreover, the microtubules are engaged with the pulling surface more to one side of the centrosome than to the other. The other side of the aster becomes engaged during the reverse swing (Figure 4B and Video S4). Similarly to a model for pronucleus oscillations in worm eggs [12], it can be observed that the distal (“plus”) ends of microtubules hardly move during the oscillation cycle. This should be attributed to the cytoplasm viscosity dampening propagation of the elastic perturbation along the microtubules from their proximal parts, which may be pulled and which are coupled to the moving centrosome. As a result, when microtubules on one side are pulled and the centrosome shifts, the proximal parts of microtubules on the opposite side will be lifted off the synaptic surface (Figure 4A and 4B and Video S4). This makes the tug of war nonlinear: whenever one side is winning, this weakens the opposing side. We ascribe to this effect the fact that our model tends to swing through the middle position. At the same time the movement appears to be limited by the deformation of the microtubules on the winning side. Their distal parts are bent against the side of the cell, and therefore the zone where they can contact the pulling surface cannot extend very close to the edge of the flat synaptic zone. Movement toward the edge therefore diminishes the pulling force. This gives the elastic relaxation of the trailing microtubules time to catch up and to bring their proximal parts in apposition with the pulling surface. At this point the microtubules that trailed are lying relatively flat on the synapse. They are therefore experiencing a pulling force that is greater than the force exerted on the microtubules which led and which are now contacting the synapse only with their highly curved parts. Movement in the reverse direction ensues (Figure 4A and Video S4). It is important to point out that while microtubule elasticity orchestrates the movement, the continued oscillations are ultimately powered by the pulling forces, which work ultimately against the energy-dissipating forces of viscous drag. The source of energy is part of the present model only by implication: it is ATP hydrolysis coupled to the working cycle of the dynein motors that are behind the pulling force in the model.

**Figure 6:**
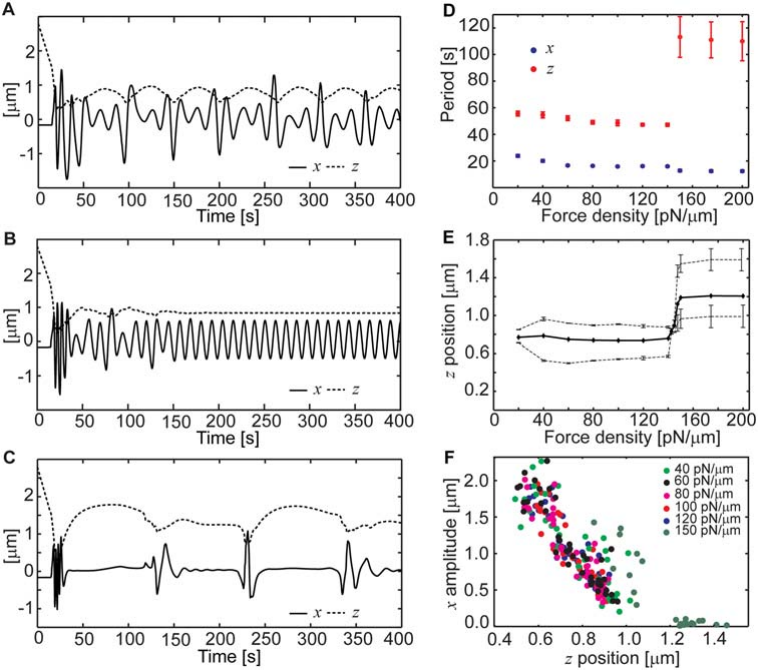
Dependence of the oscillations within the synaptic area on the pulling force density. (A–C) The three types of oscillations that are predicted correspondingly with low, intermediate, and high values of the pulling force density. The centrosome trajectory is plotted in the *x* and *z* coordinates that are the same as in Figure 4 (*x* parallel and *z* perpendicular to the synapse). In (A), the pulling force density *f* = 100 pN/µm, in (B), *f* = 143 pN/µm, and in (C), *f* = 200 pN/µm. Microtubule length, 16 µm; effective cytoplasm viscosity, 2 pN s/µm^2^. (D) The mean period of oscillations parallel and perpendicular to the synapse, as a function of the pulling force density. The error bars are S.E. (insignificant in size for most data points). Microtubule length, 16 µm; effective cytoplasm viscosity, 2 pN s/µm^2^. (E) The mean (solid line) and the characteristic minimum and maximum (dashed lines) of the centrosome distance from the synapse, as a function of the pulling force density. The minimum and maximum attained during each period were averaged over many periods to obtain the values of the minimum and maximum that are characteristic of the given force density. The error bars in this plot show the standard error associated with the statistical estimation of the characteristic minimum and maximum values. Microtubule length, 16 µm; effective cytoplasm viscosity, 2 pN s/µm^2^. (F) The peak deviation of the centrosome from the midpoint (amplitude) in oscillations parallel to the synapse (*x*) vs. the centrosome distance from the synapse *z* at the moment when the peak deviation was achieved. The datapoints are plotted for the indicated values of the pulling force density. Microtubule length, 16 µm; effective cytoplasm viscosity, 2 pN s/µm^2^.

Simulations with different pulling force densities show that the basic frequency of the oscillations is fairly insensitive to this parameter, although the overall pattern of oscillations changes abruptly when a certain value of it is crossed (Figure 6). Below approximately 140 pN/µm, the oscillations appear multiperiodic and continuous (Figure 6A). Above approximately 150 pN/µm, the oscillations are pulse-like (Figure 6C). In the relatively narrow range of pulling force densities between approximately 140 and 150 pN/µm, the oscillations are continuous and pure, i.e., they exhibit a single frequency and amplitude. Only in this narrow intermediate range does the distance of the centrosome to the synaptic plane not oscillate (Figure 6B). Based on the experimental estimate of the force that can be exerted by a single cytoplasmic dynein molecule interacting with a microtubule, 2.6 pN [13], we limit the range of the pulling force densities that are of analytical interest to between 20 and 200 pN/µm. Below this range, there will be only a few molecular motors pulling on a given microtubule, giving rise to stochasticity that our deterministic approach cannot reflect. Above this range the number will reach into the hundreds, which may not be realistic. The present model shows that within the entire range of 20–200 pN/µm, the period of oscillations parallel to the synapse remains near 15–20 s (Figure 6D). This is close to the typical frequency seen in the experimental videos [4]. This intrinsic frequency of oscillations parallel to the synapse (*x* direction) is seen in its pure form when the orthogonal (*z* direction) oscillations are absent between 140 and 150 pN/µm (Figure 6B). In the other two regimes (Figure 6A and 6C), however, measurements show that the *x*-frequency is approximately the same (Figure 6D). The period of the *z*-oscillations is also mostly insensitive to the force density, except that it is much longer for all values above 150 pN/µm than it is below 140 pN/µm (Figure 6D). In the intervening range, the *z*-oscillations are not sustained (Figure 6B), and their period, therefore, not defined. The range of the distance (*z*) of the centrosome from the synapse exhibits a similar step-like dependence on the pulling force density, collapsing fully in the narrow transition zone (Figure 6E). It can be observed that the farther away from the synapse the centrosome is at any given time, the smaller the amplitude of the movement parallel to the synapse will be. Figure 6F shows that this dependence is essentially independent of the force density and is quasi-linear. The exception to its linearity appears related to the natural limit of zero amplitude. When this limit is reached (this can happen only at high force densities), the amplitude-distance relationship exhibits a breakpoint at the axis intercept (Figure 6F). The zero amplitude of motion parallel to the synapse is observed during the intervals between the pulses, such as shown in Figure 6C. Notably, the breakpoint of the *x*-amplitude vs. *z*-position curve (Figure 6F) is near the centrosome-synapse distance of 1 µm, same as the breakpoint in the dependence of the *z*-position on the force density (Figure 6E). Close inspection shows that this transition corresponds in individual trajectories to complete but temporary loss of contact between the microtubule system as a whole and the synapse. Explanation of this phenomenon proved challenging, although it appears to arise from the viscous drag-induced “liftoff” of the microtubules that was discussed above and illustrated in Figure 4A. During particularly vigorous movement that can occur at the higher force densities, not just one side but the entire microtubule system may lose contact with the synapse (apparently due to the lift force). In the absence of the active driving force it will take the motile system considerable time to relax and contact the synapse again. These periods of time correspond to the long, high arcs of the *z*-trajectory and no *x*-movement, as seen in Figure 6C. Intuition does not appear to keep up with the complexity of the movement. It is satisfying that complexity exhibited in the simulations compares favorably with the multi-periodic and variable-amplitude movement seen in the experiments [4]. However, the mechanistic explanation of it offered by the model will be difficult to test with the existing live-cell imaging techniques, because it would depend on resolving optically the small distances around the predicted breakpoint (∼1 µm, Figure 6E and 6F).

### Inter-Synaptic Oscillations

Capacity to explain oscillations of the centrosome within a synapse is a stringent test of a mechanism proposed for centrosome polarization, and our computer simulation results indicate that the empirical hypothesis of cortical dynein pulling [4],[5] passes this test. The immunological function of the oscillations within a synapse is however unclear. (One can speculate that they might facilitate extrusion of the toxic granules.) In contrast to this uncertainty, oscillations *between two synapses* appear to be part of how a T cell engages two targets simultaneously [4], no matter how illogical this may seem from a “design” standpoint. We have therefore tested the ability of the cortical pulling mechanism to produce oscillations between two synapses as well.

**Figure 7:**
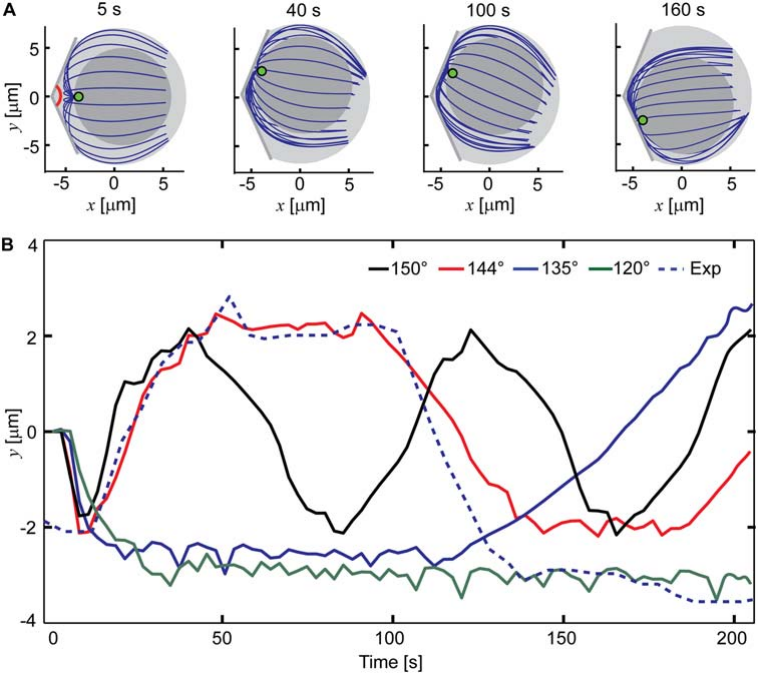
Oscillations of the centrosome between two synaptic areas. (A) Graphs of the model cell structure. The angle between the two synaptic planes is indicated by the red arc and equals 144° in this simulation. Pulling force density, 40 pN/µm; microtubule length, 16 µm; effective cytoplasm viscosity, 2 pN s/µm^2^. (B) Trajectories of the centrosome predicted for the indicated angles between the two synaptic planes. An excerpt from the experimental trajectory extracted from the supplementary video to the cited paper [4] is also shown (dashed). The illustration in (A) corresponds to the red theoretical curve. Pulling force density, 40 pN/µm; microtubule length, 16 µm; effective cytoplasm viscosity, 2 pN s/µm^2^.

Numerical solution shows that after simultaneous development of two synaptic areas on two sides of the initial centrosome position, the model centrosome goes to one of them. Which one it goes to first in our deterministic model can be decided by an otherwise insignificant deviation of the initial centrosome orientation from the middle, such as by 2°. What is important is that after pausing at the first synapse, which pause can last for a significant period of time, the model centrosome spontaneously moves to the other synapse (Figure 7A and Video S5). The cycle of movement, pause, and movement to the other synapse appears to continue indefinitely with a rather well-defined periodicity. The characteristic delay before the reverse motion is as seen in the experiments [4]. The model predicts that for the delay to take place, the angle between the two synaptic planes must be narrower than 150° (Figure 7B). The angle was indeed sharp in the experiment [4]. By only crudely adjusting the pulling force density and effective cytoplasm viscosity (to 40 pN/µm and 2 pN s/µm^2^, respectively), it is easy to reproduce with remarkable precision both the duration of the pause and the duration of the movement phase (Figure 7B). Whereas the match of the *absolute* model time scale to the experiment is a matter of (crude manual) data-fitting and therefore not particularly significant, the fact that the computed phase of pause and the computed phase of migration can have the same *relative* duration as seen in the experiment is very remarkable. The same viscosity was used in all other simulations shown, including those that, as was discussed above, reproduced closely the characteristic period of intra-synaptic oscillations independently of the pulling force density. This indicates that the deterministic mechanics of the cortical pulling mechanism may indeed account for the relevant features of centrosome motility in the T cell.

In the light of the model, the pause of the centrosome and of the associated killing apparatus next to each of the engaged targets appears to arise from the delayed relaxation of microtubules that were trailing during the last period of centrosome migration. This can be discerned by close examination of Figure 7A, and it is the same factor that leads, in the extreme, to the irreversible, ratchet-like behavior of the model cytoskeleton following very large reorientations (Figure 2). In comparison, the migration between the two synapses is medium-range, and it therefore can be reversible. Comparing it on the other hand with the relatively small-amplitude oscillations within a synapse (Figures 4 and 5), the migration of the centrosome between the synapses winds up the trailing microtubules much more around the nucleus, and it takes them longer to relax and contact the other synaptic area after the movement was limited by the deformation of the previously leading microtubules. Irrespective of these mechanistic details that are suggested by the model, it is notable that the time which the killing apparatus spends next to the given target may be determined so directly by mere elasticity of the cytoskeleton. It is equally notable that, as the model suggests, the movement of the killing apparatus to the other synapse is a direct mechanical consequence of its previous movement to the synapse where it is presently found.

Simulations in which the pulling force density at the two synapses is unequal show that the centrosome can be retained at the synapse which is the stronger, even if it visits the weaker synapse first (Figure S2). This result suggests that the preferential orientation of the centrosome and associated organelles to the stronger synapse, which was observed experimentally [14],[15] may be a limiting case of the inter-synaptic oscillations.

In summary, a purely deterministic, biomechanical model is capable of exhibiting complex, life-like centrosome movements in a conceptually simple, three-dimensional computer simulation of the dynein-pulling mechanism. Our computational results demonstrate that the origin of the strikingly animate wandering of aim in T-killer cells need not be sought necessarily in stochastic dynamics of individual molecules, or in indecision that might be exhibited by complex information processing in the T cell, or in indeterminate changes in the signaling input from the target cells. Instead, the rigorous numerical demonstration that a purely deterministic mechanical explanation exists for one of the most animate behaviors exhibited by cells suggests that similar explanations and supporting experimental evidence can be sought for other types of cell behavior that appear strikingly far from mechanistic.

## Methods

### Physical Model

#### The cell structure

The T-cell outline in our model is a sphere 14 µm in diameter. It is truncated by a plane when attachment to the target is modeled. The planar part of the model cell surface is referred to as the synapse, or synaptic surface. The entire cell surface is rigid and immobile. The nucleus is also a rigid sphere (with radius *R*_n_ = 5 µm).

#### Microtubules

There are 24 microtubules, each 25 nm in diameter. The microtubule length in the simulations was 16 µm, except where indicated otherwise. Effective (hydrodynamic, see below) microtubule diameters between 25 and 50 nm were tried, with similar results. The model microtubules are inextensible and respond elastically to flexure with the measured rigidity, *β* = 26 pN µm^2^ [16]. (Rigidities between 5 and 50 pN µm^2^ were tried, with similar results.) One end of every microtubule is clamped at the same point on the nuclear surface. This point is referred to as the centrosome. If unstressed, straight microtubules would emanate from the centrosome in a uniform conical arrangement (70° wide unless otherwise specified), but in the model they are always constrained between the nuclear and the cellular surfaces. (Unstressed microtubule divergence angles between 60 and 90° were also tested, with similar results—see Figure S3.)

#### Model initialization

Elastic relaxation of microtubules coupled with the nucleus inside the spherical cell outline comes to a static equilibrium, which is the initial condition for the dynamic simulations. A simulation is begun by intruding the truncating plane at a constant speed into the cell over 25 s to a point where it truncates the sphere by 2 µm. The cell volume is kept constant by a corresponding (minor) increase of the radius of the round part. (Intrusion depths between 1.8 µm and 2.6 µm were tried, with similar results.)

#### Pulling

At all times after the beginning of the simulation, microtubules can slide according to the following rules: When part of a microtubule is within a small distance (15 nm) from the synaptic surface, force is exerted on that part of the microtubule. (Contact distances between 10 and 100 nm were also tried, with qualitatively similar results.) The force is tangential to the microtubule and directed towards its distal (“plus”) end. There is a constant magnitude of force exerted per unit length of the microtubule within the specified contact distance, which is referred to as the pulling force density. These rules would describe microtubules coming in contact with the cell cortex on which dynein molecules are anchored at a certain spatial density, if the dynein is activated upon the synapse formation as hypothesized [4],[5]. The pulling force density was 40 pN/µm in most simulations; the specific values are indicated in the corresponding figure legends.

#### Drag

Movement of the microtubules and nucleus is opposed by viscous drag (overdamped motion, see below). We chose the effective viscosity of the cytoplasm so as to reproduce the characteristic speed of the centrosome movements in T cells. To arrive at this value, we proceeded from the drag coefficient value that was similarly chosen in a comparable type of model that approximated well the movements during cell division [10]. Viscosity was estimated from the consideration that nucleus in our model would have the same translational drag coefficient. Our best-fit value of 2 pN s/µm^2^, which was used in the simulations shown, turned out to be four times lower. (Viscosities between 0.1 and 8 pN s/µm^2^ produced qualitatively similar results.)

#### Dynamic instability

As explained above, the length of the each microtubule was kept constant, with the exception of special additional modeling cases. In these special additional simulations we tested the impact of dynamic instability (stochastic changes of microtubule length [6],[7]) on the otherwise deterministic dynamics of our model. When dynamic instability was incorporated in the model, it was assumed that a) length fluctuations in individual microtubules were independent, and b) the variance in microtubule length increased linearly with time, with the apparent diffusion coefficient of microtubule length (by definition, half the rate of variance growth) of 3.33 µm^2^/min. This value was chosen to lie between those measured in PtK [17] and melanophore cells [18]. It was shown previously that dynamic instability can be adequately described on the cell scale by the rate at which the length variance increases with time regardless of the actual kinetic complexity (diffusion approximation of dynamic instability as a stochastic process [19]).

### Numerical Solution

#### Microtubules

Microtubules are represented numerically as chains of straight segments that approximate the centerlines of the microtubules. Each microtubule was approximated with 32 segments of equal equilibrium length.

#### Inextensibility

The essential inextensibility of microtubules is implemented by assigning a high Hookean spring constant to the segments. The value of 2000 pN/µm for this constant results in a force restoring the length of the segment which becomes much larger than other typical forces in the model before the segment length change becomes noticeable. In effect, therefore, the microtubules in our numerical model are inextensible and incompressible.

#### Bending

The restoring forces resulting from flexural rigidity of a bent microtubule were calculated in a slightly more generalized way compared to the previously developed mitotic spindle model [20]. Let us number the segment joints (“nodes”) in a microtubule sequentially by the index *i*, and denote the Cartesian coordinates of the *i*-th node as **x** *_i_*. We calculate the microtubule curvature at *i*, *κ_i_*, approximately as the angle between the directions of the two segments joined at *i*, divided by the average of their lengths. Implementing the torque *βκ_i_* through forces exerted at the neighboring nodes, and preserving the overall force balance, we calculate the force exerted on node *i* which reflects the microtubule bending stiffness as
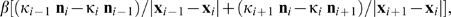
where **n** *_i_* is the approximated inward normal to the microtubule at *i*. **n** *_i_* was calculated by considering the plane determined by **x** *_i−_*_1_, **x** *_i_*, and **x** *_i+_*_1_. There are two co-planar unit vectors, **u** and **v**, which are perpendicular to segments 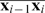 and 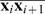, respectively. Furthermore, **u** and 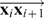, and **v** and 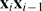, form acute angles. **n** *_i_* is the normalized average of **u** and **v**.

#### Clamping

To implement our assumption that the microtubule ends are not merely anchored at the centrosome, but clamped there, the above numerical treatment of microtubule bending was applied not only to flexure between actual microtubule segments, but also to the deflection of the first proximal microtubule segment from the direction fixed with respect to the nucleus in the manner described among the physical assumptions.

#### Impenetrability

To implement impenetrability of the cell outline to the microtubules, whenever a node on a microtubule approaches the cell outline closer than the microtubule radius, a reaction force is exerted on that node. The direction of this force is inward normal to the cell outline at the point of its contact with the microtubule. The force magnitude is calculated so that with the drag coefficient associated with the node (explained below), the node will just stop violating the impenetrability condition at the next time step. This definition results in maximally precise implementation of impenetrability without causing numerical instability in our time-stepping scheme. Nucleus violating the cell outline is treated in the same way. Any noticeable penetration of the nuclear volume by microtubules is prevented similarly, with the action and reaction forces similarly calculated and exerted on the nucleus and the microtubule node.

#### Drag

Viscous drag on the nucleus and microtubules was calculated using the same values of effective viscosity of the cytoplasm, *η*, chosen as explained among the physical assumptions. We used the widely accepted approximations for the drag coefficient: With a translational velocity **v** and rotational velocity **ω** of the nucleus, the viscous drag and torque on the nucleus were calculated as −6π*ηR*_n_ **v** and −8π*ηR*_n_^3^ **ω**, respectively, where *R*_n_ is the radius of the nucleus as specified above. The distribution of the drag force along a microtubule was calculated using the numerical representation of microtubules as segmented chains, which was described above. The velocity of a microtubule node **v** was decomposed into the components locally normal (**v**_⊥_) and tangential (**v**_∥_) to the microtubule. The drag force on the node was then calculated as −2π*η*(**v**_∥_+2**v**_⊥_)*l*/log(*l*/2*r*), where *r* is the microtubule radius as specified above, and *l* is the resting length of the microtubule segment, per the above numerical specifications.

#### The time-stepping scheme

The forward Euler method [21] was used to integrate the motion of the microtubules and nucleus. The instantaneous velocities were derived from the condition whereby all forces and torques exerted on the microtubules and nucleus are balanced by the viscous drag force that resists their motion through the cytoplasm. (I.e., overdamped, zero-Reynolds number conditions were assumed, as in other models of intracellular movement of the microtubule cytoskeleton and nucleus, e.g., as in refs. [10]–[12].) The time step used was Δ*t* = 0.0002 s. The following presents more details on the calculations used to update the positions of the microtubules and nucleus during a time step. First we consider a microtubule node (see above) at position **x** in the three-dimensional Cartesian coordinates. (This does not apply to the most proximal, or centrosome, node on each microtubule, which is in fact part of the rigid-body nucleus.) Forces acting on this node are calculated as detailed in the corresponding subsections above. These forces arise from (1) bending, (2) inextensibility, (3) impenetrability of boundaries, and (4) pulling at the synapse. We denote the sum of these forces by **F**. This force will be balanced by viscous drag force. Since the drag coefficient of the microtubule segment represented by the node depends on the direction of movement, in the following calculation **F** is decomposed into the components that are locally parallel to the microtubule, **F**_∥_, and orthogonal to it, **F**_⊥_. When divided by the appropriate drag coefficient, these components of force will yield the corresponding components of the node velocity. Therefore, using the widely accepted approximations for the drag coefficient of a cylinder (see above), the position of the node should be updated as follows:
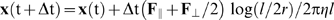



The following procedure is used to update the position and orientation of the nucleus (together with the centrosome node and the vectors representing the unstressed microtubule emanation directions, see above). The total force **F**_n_ applied to the nucleus arises from the conditions of impenetrability (see above) and from the forces applied to the centrosome node. The latter are calculated the same way as for the generic microtubule node (see above). The position of the nucleus center **x**_n_ is updated as follows, in accordance with the above formula for drag and with the forward Euler method:
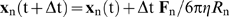



The total force moment is also calculated. It arises only from the forces applied to the centrosome, at the distance *R*_n_ from the nucleus center. (All other forces exerted on the nucleus arise from the impenetrability conditions and are therefore directed to the nucleus center.) Torque balance with the drag force determines the angular velocity **ω** of the nucleus:
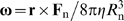
where **r** is the vector from the centrosome to the nuclear center. The obtained instantaneous value of **ω** is then used to calculate the displacement of the centrosome node that is due to the nucleus rotation during the time step Δt, and to rotate the vectors of the unstressed microtubule emanation directions.

#### Dynamic instability

In those special simulations that incorporated the dynamic instability of microtubules, the following algorithm was used. At each time step, a probabilistic decision was made independently for each microtubule, whether to add a new segment to its free end, remove the end segment, or do nothing. A pseudorandom number was generated from a uniform distribution between 0 and 1. If the number was smaller than a small parameter *p*, a segment was added. If the random number was larger than 1−*p*, a segment was removed. Otherwise, no changes were made. The nondimensional parameter *p* was chosen so that when multiplied by the dimensionality constant *l* ^2^/Δ*t*, where *l* is the unstressed length of a microtubule segment and Δ*t* is the time step size, it would be equal to *D*, the apparent diffusion coefficient of microtubule length (see physical assumptions above). This algorithm is a discrete implementation of a diffusion-type stochastic process that is used to approximate dynamic instability of microtubules [19]. New segments added were parallel to the segment that was previously the end segment of the microtubule. When this resulted in a violation of any of the impermeable boundaries, the same rules applied to the new segment as to any microtubule segment that violated the boundaries (see above).

## Supporting Information

Figure S1 Centrosome reorientation that is caused by cell outline deformation alone, in the absence of the pulling force. Centrosome orientation is measured as the angle formed by the vector drawn from the nucleus center to the centrosome and by the outward normal to the synapse. (I.e. 0 means centrosome pointing at the synapse and 180°, at the opposite side of the cell.) The thin straight line is drawn for reference; it indicates where the simulation results would lie if there would be no reorientation. To generate these results, the pulling force density in the model was set to zero. Microtubule length, 16 µm; effective cytoplasm viscosity, 2 pN s/µm^2^.
(0.45 MB TIF)

Figure S2 Stabilization of the centrosome next to the stronger synapse. Plotting conventions are as in Figure 7. The simulations were set up as in Figure 7A, except for a slightly larger symmetry-breaking tilt of both synaptic planes (5°). In the simulation shown by the blue curve, pulling force on both synapses was 40 pN/µm, and symmetric oscillations between the synapses developed. In the simulation shown by the red curve, one synapse had pulling force density 4 pN/µm, the other 80 pN/µm. In both simulations, microtubule length was 16 µm and effective cytoplasm viscosity, 2 pN s/µm^2^. The centrosome migration from the weaker to the stronger synapse appeared irreversible. The large strength difference was tested because in the experiments that inspired this test, the antigen load of the target cells differed by a factor of ∼1000 [14],[15].
(0.76 MB TIF)

Figure S3 Sensitivity of the model to the value of the unstressed microtubule divergence angle. (A) Centrosome reorientation plotted for the indicated values of the unstressed microtubule divergence angle. The ordinate is the angle formed by the vector drawn from the nucleus center to the centrosome and the outward normal to the synapse. (The 90° starting angle means that centrosome in these simulations was initially on the side of the cell with respect to the synapse.) The plots illustrate relative insensitivity of the reorientation trajectory to the divergence angle. Pulling force density, 40 pN/µm; microtubule length, 16 µm; effective cytoplasm viscosity, 2 pN s/µm^2^. (B) Intra-synaptic oscillations plotted for the indicated values of the unstressed microtubule divergence angle. x is the coordinate axis directed across the synapse, as shown in Figure 4A. The plots illustrate relative insensitivity of the oscillation trajectory to the divergence angle. Pulling force density, 20 pN/µm; microtubule length, 16 µm; effective cytoplasm viscosity, 2 pN s/µm^2^.
(0.99 MB TIF)

Video S1 Reorientation of the centrosome to the synapse. This video corresponds to the first part of Figure 2 and follows the graphical conventions in that figure. Pulling force density, 40 pN/µm; microtubule length, 16 µm; effective cytoplasm viscosity, 2 pN s/µm^2^.
(3.37 MB MOV)

Video S2 Reorientation followed by oscillations of the centrosome. Pulling force density, 20 pN/µm; microtubule length, 16 µm; effective cytoplasm viscosity, 2 pN s/µm^2^.
(6.06 MB MOV)

Video S3 Oscillations in a model which in addition to our usual assumptions incorporates also dynamic instability of microtubules and a ring-shaped pulling surface. The area of the synaptic surface where pulling is activated is shown in black, the parts of the synapse that are inactive as far as pulling are shown in white. The cell surface is cut out for a clearer view (only in graphics, not in actual simulation). Pulling force density, 36 pN/µm; starting microtubule length, 16 µm; effective cytoplasm viscosity, 2 pN s/µm^2^.
(1.33 MB MOV)

Video S4 Oscillations in detail. This video is an animation of Figure 4 and follows its graphical conventions. On the left is a side view of the entire model and on the right is the bottom view (looking through the synaptic surface but not showing this surface). In the view on the right, the parts of the microtubules that are in close contact with the inner synaptic surface and therefore experience pulling are highlighted. The video shows two oscillation cycles before it ends. Pulling force density, 20 pN/µm; microtubule length, 16 µm; effective cytoplasm viscosity, 2 pN s/µm^2^.
(8.61 MB MOV)

Video S5 Oscillations between two synapses. This video is an animation of the same simulation that is shown in Figure 7A. Pulling force density, 40 pN/µm; microtubule length, 16 µm; effective cytoplasm viscosity, 2 pN s/µm^2^.
(5.51 MB MOV)
