# Supplementary figures and images for: Deterministic Mechanical Model of T-Killer Cell Polarization Reproduces the Wandering of Aim between Simultaneously Engaged Targets
Source: PLoS Comput Biol. 2009 Jan 9;5(1):e1000260. doi: 10.1371/journal.pcbi.1000260 (PMC2603019; doi:10.1371/journal.pcbi.1000260)

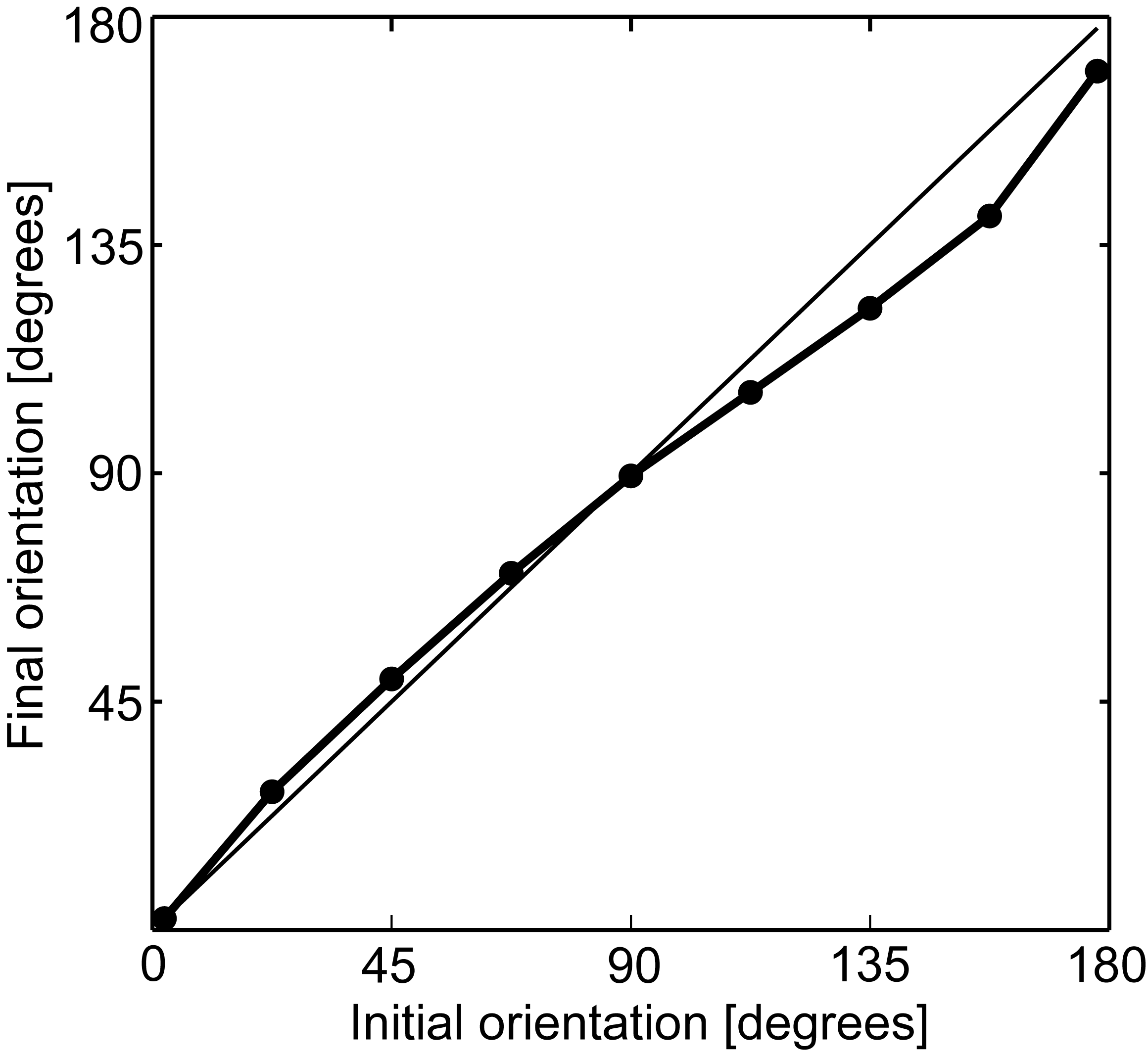

Supplement: Figure S1 — Centrosome reorientation that is caused by cell outline deformation alone, in the absence of the pulling force. Centrosome orientation is measured as the angle formed by the vector drawn from the nucleus center to the centrosome and by the outward normal to the synapse. (I.e. 0 means centrosome pointing at the synapse and 180°, at the opposite side of the cell.) The thin straight line is drawn for reference; it indicates where the simulation results would lie if there would be no reorientation. To generate these results, the pulling force density in the model was set to zero. Microtubule length, 16 µm; effective cytoplasm viscosity, 2 pN s/µm2. (0.45 MB TIF) [file pcbi.1000260.s001.tif]

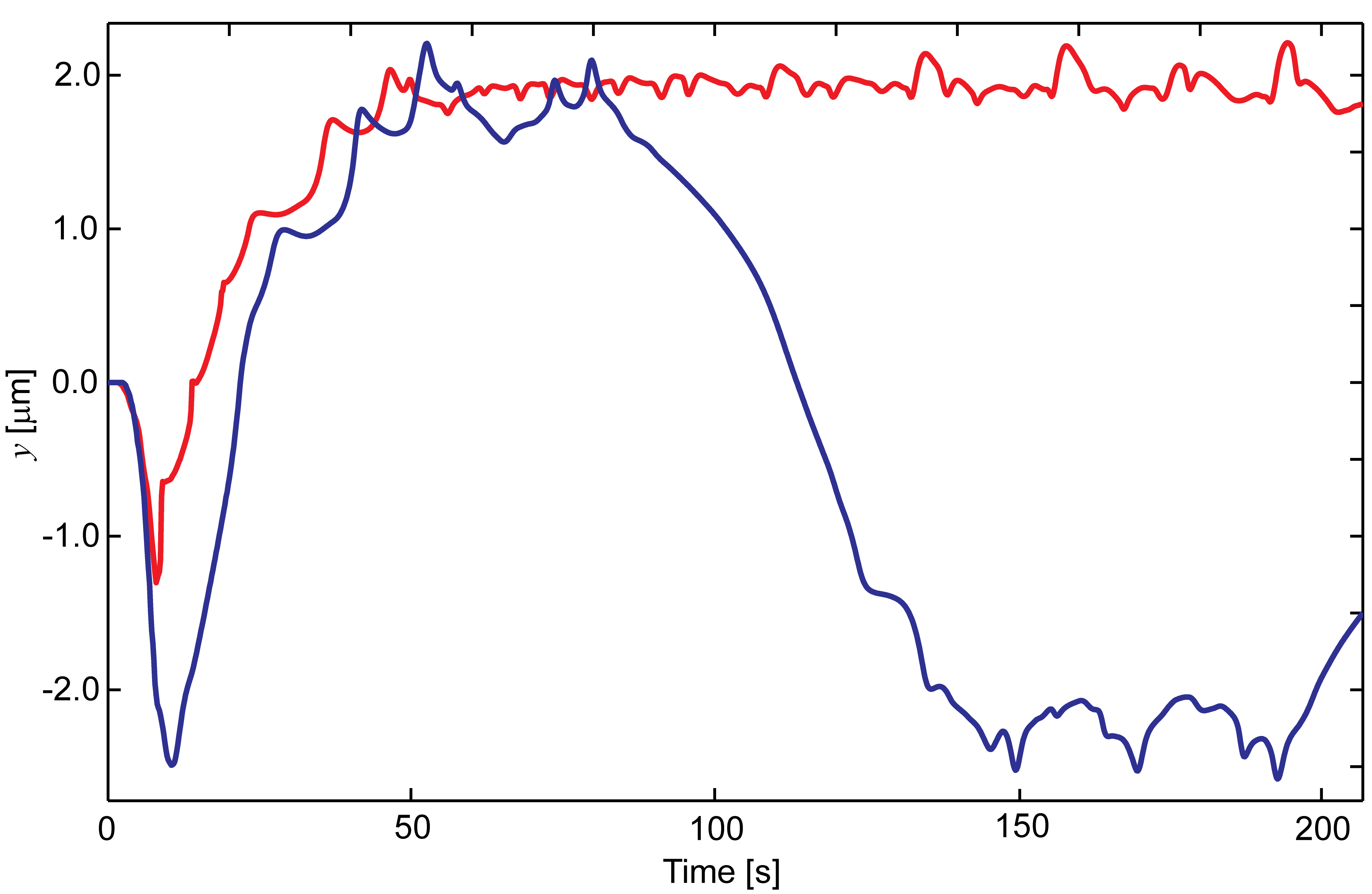

Supplement: Figure S2 — Stabilization of the centrosome next to the stronger synapse. Plotting conventions are as in Figure 7. The simulations were set up as in Figure 7A, except for a slightly larger symmetry-breaking tilt of both synaptic planes (5°). In the simulation shown by the blue curve, pulling force on both synapses was 40 pN/µm, and symmetric oscillations between the synapses developed. In the simulation shown by the red curve, one synapse had pulling force density 4 pN/µm, the other 80 pN/µm. In both simulations, microtubule length was 16 µm and effective cytoplasm viscosity, 2 pN s/µm2. The centrosome migration from the weaker to the stronger synapse appeared irreversible. The large strength difference was tested because in the experiments that inspired this test, the antigen load of the target cells differed by a factor of ∼1000 [14],[15]. (0.76 MB TIF) [file pcbi.1000260.s002.tif]

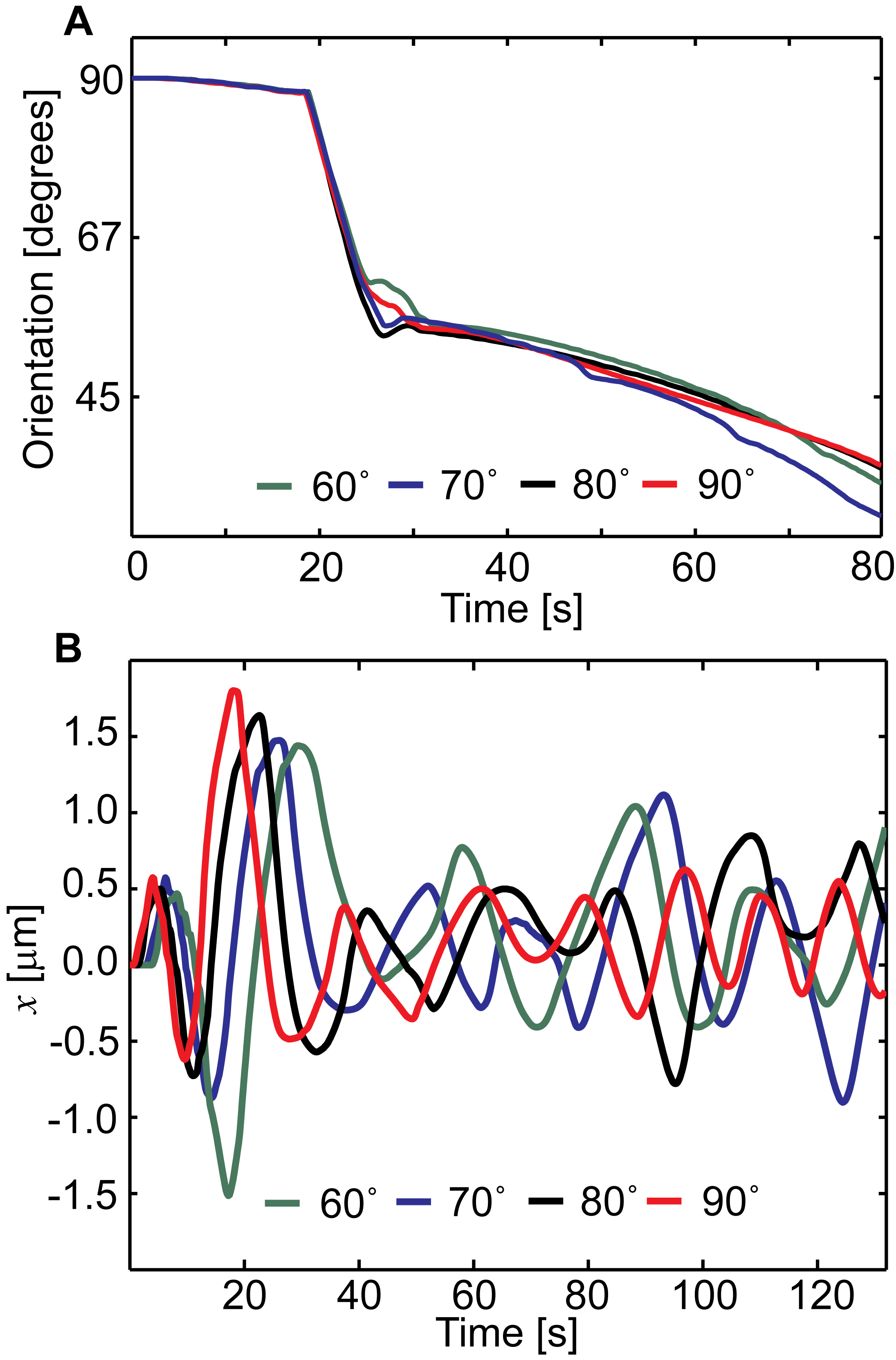

Supplement: Figure S3 — Sensitivity of the model to the value of the unstressed microtubule divergence angle. (A) Centrosome reorientation plotted for the indicated values of the unstressed microtubule divergence angle. The ordinate is the angle formed by the vector drawn from the nucleus center to the centrosome and the outward normal to the synapse. (The 90° starting angle means that centrosome in these simulations was initially on the side of the cell with respect to the synapse.) The plots illustrate relative insensitivity of the reorientation trajectory to the divergence angle. Pulling force density, 40 pN/µm; microtubule length, 16 µm; effective cytoplasm viscosity, 2 pN s/µm2. (B) Intra-synaptic oscillations plotted for the indicated values of the unstressed microtubule divergence angle. x is the coordinate axis directed across the synapse, as shown in Figure 4A. The plots illustrate relative insensitivity of the oscillation trajectory to the divergence angle. Pulling force density, 20 pN/µm; microtubule length, 16 µm; effective cytoplasm viscosity, 2 pN s/µm2. (0.99 MB TIF) [file pcbi.1000260.s003.tif]
